# Annotations

**Published:** 1895-12-07

**Authors:** 


### Annotations.

### The Pasteurisation of Milk.

Those who were present, as the writer was, at the annual meeting of the British Medical Association in Edinburgh, about twenty years ago, when Sir Joseph Lister demonstrated to an enthusiastic audience the germ theory of putrefaction, and showed a convincing series of test tubes charged with milk in various stages of putrefactive progress, will readily comprehend what is meant by the Pasteurisation of milk. The milk of the cow is one of the most variously useful of all human foods, and its preservation in food value and flavour is a desideratum of first-rate importance to men and women in every part of the world. Milk is a substance for which certain putrefactive germs seem to have a special liking. Everybody knows how promptly "sourness" is set up in it during very hot or very damp weather. This is entirely due to the entrance of germs from the air or other external source. By merely excluding the air from milk, that is, by drawing it from the udder in such a way as to secure its passage into a hermetically sealed tube without contact with air or other external agent, milk may be kept sweet and wholesome practically for ever. The object of the Pasteurisation of milk, however, is not to draw it into hermetically sealed vessels, for that would in practice be impossible, but to render it, by means of scientifically applied heat, incapable of putrefaction in the ordinary way. It is much to be desired that this beneficial application of one of Pasteur's earlier discoveries may prove commercially successful, for that the theory upon which the Pasteurians are proceeding is sound cannot be questioned.

### The Medical Council and the Medical Profession.

Many are the cries sent up by the members of the medical profession who wish to see some return for the £5 paid by them to the Council for the honour of being entered on their Register; and we fancy that the discontented ones, consisting largely of members of the Council of the British Medical Association, will derive but scant encouragement from the remarks of Sir Richard Quain, President of the General Medical Council, on the occasion of its opening meeting. What he took special pains to point out was that the Medical Council exists for the protection of the public, and only incidentally for the protection of the profession. He says the Council was "created primarily in the interests of the public to secure for that public competent professional aid. . . . The protection of the interests of the public is the first duty assigned to the Council." The Council has "had assigned to it the duty of forming an accurate Register of competent practitioners, and to keep that Register free from all unworthy elements. The Council has also the duty of preparing and publishing the British Pharmacopœia." That seems to be all. As for undertaking other duties, such as "taking cognisance of matters of professional etiquette and improper conduct and action" of members of the profession, the Council will have none of it. Here we have, put into rather plainer terms than usual, what might be looked upon as the essence of a very pretty grievance, and it is not difficult to sympathise with the doctors who bewail the loss of their £5, and look with envy upon the lawyers, protected as they seem to be by the penalties which fall on all who poach on their preserves. It certainly is hard that the doctors should get no better protection from quacks than they do; but that, we fear, is the outcome of the law rather than of any special malice on the part of the General Medical Council, and Sir Richard Quain certainly scored a point when he showed that, as against the £5 paid by doctors, solicitors, before being admitted to practise, have to pay stamp duties to the Inland Revenue authorities amounting to £110, and also an annual fee for a licence to practise in London of nine guineas each, and to practise in the country of six guineas each. It was also pointed out with especial unction that over the expenditure of these fees the lawyers have no control whatever, while on the Medical Council there are at least *some* direct representatives. Thus the doctors "should not be discontented with their position as compared with that of the legal profession, so far as the payment of fees and the control of the expenditure is concerned." Now, then, comes the great fight! The British Medical Association nails its colours to the mast, and declares for direct representation. Meanwhile the public smiles, and asks: Why should these doctors want to be more largely represented on a body whose primary function it is to keep them themselves up to the mark? It is a nice problem, and it is not difficult to see that it has two sides.

### The Health Work of a City Company.

The extraordinary interest now taken in questions of public and private health by every class of the community is shown by the almost unexampled gathering at the Saddlers' Hall on Wednesday in connection with the Plumbers' Company. There were present the Chief Magistrates or their representatives of various cities, and an assemblage of all sorts and conditions of sanitarians, such as it seemed almost impossible to get together under one roof. And probably this extraordinary assembly would not have been got together if it had not been for that most irresistible of all attractions to the genuine Englishman—a good dinner. The modern plumber, it seems, is a kind of half sanitary engineer and half doctor. He must have such a knowledge of smells and tastes, with their good or evil effects upon the human organism, as the doctor possesses; and such a capacity for safely conveying away all waste and deleterious matter as the practical engineer can boast of. Thus educated and equipped, he can convert the most unwholesome of dwellings and public places into perfect Arcadias for sweetness, provided you give him a plentiful supply of money. It is the worthy ambition of the Plumbers' Company so to utilise its funds—not very large funds, by the way—as to provide for every town or village in Great Britain as many thoroughly competent plumbers as the necessities of the country demand. To secure the registration which they ask for they require an Act or Acts of Parliament; and everybody who has the least comprehension of the almost infinite importance of pure water, efficient drainage, and a sweet atmosphere, must wish them success in their efforts to obtain the legislation they demand.

